# Nutrient availability of roughages in isocaloric and isonitrogenous diets alters the bacterial networks in the whole gastrointestinal tract of Hu sheep

**DOI:** 10.1186/s12866-023-02814-z

**Published:** 2023-03-15

**Authors:** Yuqi Li, Jian Gao, Yihan Xue, Ruolin Sun, Xiaoni Sun, Zhanying Sun, Suozhu Liu, Zhankun Tan, Weiyun Zhu, Yanfen Cheng

**Affiliations:** 1 Laboratory of Gastrointestinal Microbiology, National Center for International Research On Animal Gut Nutrition, Nanjing Agricultural University, Nanjing, 210095 China; 2 College of Animal Science, Tibet Agricultural and Animal Husbandry University, Nyingchi, 860000 China

**Keywords:** Wheat straw, Roughage, Gastrointestinal tract, Hu sheep, Microbiota

## Abstract

**Background:**

The nutrient availability of roughages could affect the dietary utilization efficiency of ruminants even in isocaloric and isonitrogenous diets. Here, we analyzed the bacterial composition and their metabolic pathways in the gastrointestinal tracts (GITs) of Hu sheep fed with wheat straw (WS) instead of alfalfa (AL) in isocaloric and isonitrogenous diets, trying to explore the reasons from the perspective of GITs bacterial network structure changes.

**Results:**

We employed 16S rRNA gene sequencing in combination with the Kruskal–Wallis test, Spearman correlation analysis, and other statistical methods to describe the microbiota composition in the GITs of Hu sheep. The results showed after the roughage was replaced from AL to WS, the most positive response occurred in the rumen microbiota, resulting in a more obvious microbiological and functional redundancy phenomenon. Whereas extended biogeographic studies of the GITs bacterial community found opposite results for the hindgut microbiota and metabolism networks compared to the forestomach. The abundance of fiber-degrading bacteria such as *Prevotella*, *Oscillospiraceae NK4A214 group*, and *Treponema* was significantly increased in GITs, but low-efficiency crude fiber degradation inhibited energy use efficiency, the pentose phosphate pathway, gluconeogenesis, and volatile acid synthesis. In addition, dietary shifting from AL to WS decreased the abundance of beneficial bacteria such as the *Lachnospiraceae NK3A20 group* and *Alistipes*, thereby enhancing the underlying inflammatory response.

**Conclusions:**

These findings suggest that feeding untreated WS affected the structure and function of the bacterial network in the GITs due to limited total digestible nutrients, and in particular increases the complexity of the rumen bacterial network, and limit the abundance of bacteria involved in the crude fiber degradation in the hindgut.

**Supplementary Information:**

The online version contains supplementary material available at 10.1186/s12866-023-02814-z.

## Background

Domesticated ruminants are crucial sources of animal meat and dairy products for humans, and their population would further increase with discretionary incomes and urbanization [1]. Roughages are important feeds for ruminants to provide the carbon skeleton of energy and amino acids for rumen microbes [2], maintain rumen health [3], and control feed intake through physical filling [4]. Within the roughages, alfalfa (*Medicago sativa L*.) is a high-quality legume forage rich in crude protein and a variety of growth-promoting factors [5] and easy to digest [6], which makes it to be a worldwide popular forage. However, its production could not meet the needs of animal husbandry and the amount of imported alfalfa is increasing in China. Recently, the increased price of alfalfa also damages the profits of the animal husbandry industry in China [7]. Thus, it's important to seek alternative roughage for alfalfa to meet the demand for roughage and reduce the feed costs for the ruminant industry. Wheat straw (WS) is the second most abundant agricultural residue in China [8]. Based on the high yield and low price of straws, WS could be the alternative roughage for ruminants to alleviate the shortage of high-quality forages such as alfalfa hay (AL) [9–12]. Previous studies reported that WS rich in effective neutral detergent fiber could maintain rumen health in ruminants fed high-grain diets [13]. The feed resources containing low nutrient density such as WS could also stimulate feed intake in cows during the dry period and promote a smooth transition of the cows to lactation [14].

However, the lignocellulosic structures in WS would cause a marked decrease in animal performance (e.g., feed intake and body weight decrease) and an increase in feed conversion ratio in ruminants [9, 10] compared with alfalfa, even under the isocaloric and isonitrogenous diets. The reason could be that lower contents of total digestible nutrients in WS would affect the composition and colonization of microbiota in the gastrointestinal tract (GITs) of ruminants. When untreated straw was used as forage, its indigestible fibrous structure increased the diversity and richness of the rumen bacterial community together with the relative abundance of Bacteroidetes, especially *Prevotellaceae*. However, it would reduce the relative abundance of *Firmicutes* and depress microbial protein synthesis resulting in the decline of nutrient utilization in the rumen, even under isocaloric and isonitrogenous diets [9, 15, 16]. Similar results were found in the in vitro rumen fermentation [17]. The changed bacterial community in the rumen ecosystem caused by the nutrient availability of roughages would further alter the co-abundances among microbes or metabolic pathways [18], due to the redundancies of microbial functions for complex carbohydrates in ruminants [19].

Current research on ruminant GITs' microbial responses to straw has mainly focused on the microbiota within the rumen which accounts for 60% to 80% of the total volume of the digestive system [20]. Some study suggests that the hindgut (cecum, colon) of ruminants is also rich in fiber-degrading microbiota [21]. However, there are few studies on the response of other parts of GITs microbiota to WS except the rumen. Thus, we hypothesized that the effect of dietary shifting of roughages from AL to WS on gastrointestinal microbiota was not limited to the rumen, but extended to the entire GITs in ruminants. This study aimed to investigate the dietary shifting of roughages from AL to WS on the bacterial community and their metabolic functions in different parts of GITs in sheep using the 16S rRNA sequencing and function predictions. Expanded biogeographic studies of the GITs in ruminants will help to profile further the microbial response to low-quality straws and increase the utilization of straws in ruminants to alleviate the shortage of high-quality roughages.

## Materials and methods

### Ethical approval

This experiment was approved by the Animal Care and Use Committee of Nanjing Agricultural University [SYXK (SU) 2021–0086]. All procedures followed the regulations of the Measures for the Management of Experimental Animals in Jiangsu province [22] and the ARRIVE guidelines (https://arriveguidelines.org/arrive-guidelines).

### Experimental design and sampling

Twelve healthy male Hu sheep (5 months old, 40 ± 2 kg liveweight) selected as the experimental animals were purchased from the Institute of Animal Husbandry, Jiangsu Academy of Agricultural Sciences (Nanjing, China). Each animal was house-fed in a single cage and had free access to drinking water and feed. Table 1 showed the ingredients and nutrients of two experimental rations prepared according to the nutrient requirements of meat-type sheep and goats (NY/T 816–2021) [23]. During adaptation (30 days), all sheep were randomly divided into two groups and fed the alfalfa-based diet. After adaption, two groups of sheep were fed the alfalfa-based diet (AL group, *n* = 6) and wheat straw-based diet (WS group, *n* = 6) at 8:00 h and 17:00 h each day for 28 days. On day 28, all sheep were slaughtered before morning feeding, and the different segments of GITs were ligated, severed, and weighed including the rumen, reticulum, omasum, abomasum, jejunum, cecum, colon, and rectum. The GITs digesta and feces were collected and stored at -80 °C for further DNA extraction.

**Table 1:** Feed ingredient and nutrient composition

Items	Treatments	
	Alfalfa	Wheat straw
Ingredients, % of DM		
Corn	30.00	34.50
Bean pulp	0.00	20.50
Wheat straw^a^	0.00	40.00
Alfalfa hay	40.00	0.00
Wheat bran	24.50	1.00
Zeolite	2.50	0.00
Limostone	0.00	1.00
Premix^b^	2.50	2.50
NaHCO_3_	0.50	0.50
Nutritional levels, % of DM, unless otherwise noted		
Metabolizable Energy, MJ/kg of DM	8.21	8.14
Digestible energy, MJ/kg of DM	9.60	9.58
Crude protein	12.80	12.79
Crude fiber	12.76	14.59
Ether extract	2.51	2.28
Calcium	0.53	0.48
Phosphorus	0.46	0.25

^a^: The wheat variety is “Shenmai 118”, ^b^: Premix contains vitamin A, 70 KIU; vitamin D_3_, 8 KIU; vitamin E, 50 mg; CuSO_4_, 325 mg; ZnSO_4_, 750 mg; FeSO_4_, 550 mg; and MnSO_4_, 600 mg per kg

### DNA extraction and 16S rRNA sequencing of bacterial community

Total genomic DNA of microbes in digesta samples was extracted with a phenol/chloroform/isoamyl alcohol solution using a microbead beater (Biospec Products, OK, USA) as the method described by Sun et al*.* [24]. The solution was then precipitated with ethanol, and the precipitate was dissolved in 50 ml of pH 8.0 Tris–EDTA buffer. Quality and concentration of microbial DNA were determined by a Nanodrop 2000 spectrophotometer (Thermo Fisher Scientific, MA, USA) and then stored at -20 °C for further analysis. The V4 region of bacterial 16S rRNA was amplified using the degenerate primers 515 F (5′-GTGCCAGCMGCCGCGTAA-3′) and 806 R (5′-GGACTACHVGGTWTCTAAT-3′) [25]. Both forward and reverse primers were tagged with Illumina adapters, pads, and linker sequences. The PCR reaction was conducted in a 50 μL reaction containing 30 ng of template, fusion PCR primers, and PCR master mix. The PCR cycling conditions were as follows: 95 °C for 3 min, 30 cycles of 95 °C for 45 s, 56 °C for 45 s, 72 °C for 45 s, and final extension for 10 min at 72 °C. The PCR products were purified using Agencourt AMPure XP beads and eluted in an elution buffer. Libraries were characterized by an Agilent Technologies 2100 Bioanalyzer. By the standard protocol of BGI (Shenzhen, China), the validated amplicon libraries were sequenced on the Illumina HiSeq 2500 platform using paired-end sequencing reads (2 × 250 bp).

### Bioinformatics and Diversity Analyses

Raw reads were initially filtered using the fastp (v0.23.2) [26] with the following parameters: -q 30 -l 100, and then the trimmed reads were imported into QIIME 2 (v2022.2) [27]. Amplicon sequence variants (ASVs) were identified using the DADA2 tutorial (https://benjjneb.github.io/dada2/tutorial.html) after the removal of chimeras and filtration of low-quality sequences. The ASVs in at least 2 samples were retained to avoid accidental factors. Microbial taxonomy was aligned and annotated with the SILVA database (v138) [28]. Alpha and beta diversities were estimated using the q2-diversity method with a sample depth of 30,500. Linear discriminant analysis (LDA) effect size (LEfSe) analysis was used to identify the differential bacteria and pathways between treatments [29]. Furthermore, the functional profiles of microbial communities were predicted using the Phylogenetic Investigation of Communities by Reconstruction of Unobserved States (PICRUSt2) (https://github.com/picrust/picrust2) [30].

### Statistical analyses and visualization

Concretely, The R software (v4.1.3) was employed for microbial community statistical analysis (Kruskal–Wallis test) and data visualization. The correlation networks among bacteria communities or metabolic pathways at level-3 Kyoto Encyclopedia of Genes and Genomes (KEGG) [31–33] were analyzed using the Spearman correlation analysis and visualized with GraphPad Prism (v8.0). Significant correlations with coefficient > 0.8 and *P* < 0.05 were visualized on the Gephi platform (v0.9.7). All data were shown as mean ± standard error of the mean (SEM), *P* < 0.05 or FDR adjusted *P* < 0.05 was considered statistically significant.

## Results

### GITs weight, bacterial richness and diversity

**Fig. 1:**
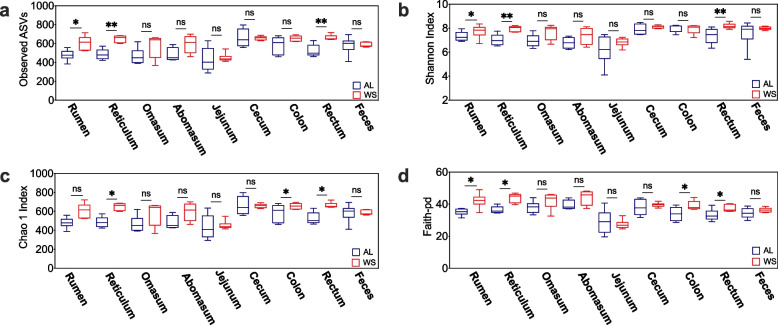
Comparison of alpha diversity between treatments. **a** observed ASVs index; **b** Chao 1 index; **c** Shannon index; **d** Faith-pd. WS, wheat straw group; AL, alfalfa group. *: *P* < 0.05; **: *P* < 0.01; ns: no significance

Dietary shifting of roughages from AL to WS caused a 59.4% increase in the full weight of the sheep rumen (*P* < 0.05, Table S1), whereas weights of other parts in GITs of sheep showed no difference between treatments (*P* > 0.10). For the bacteria community in GITs, Fig. S1 showed that the rarefaction curves stabilized when the sequences exceeded 30,000. The sequence coverage in all samples exceeded 99.9%, indicating a good sequencing depth for the further investigation of microbiota. Shifting roughages from AL to WS significantly increased the observed ASVs number, the faith-pd and the Shannon indices in the rumen, reticulum, and rectum, the Chao1 indices in the reticulum, colon, and rectum, and the faith-pd indices in the colon (*P* < 0.05) (Fig. 1).

**Fig. 2:**
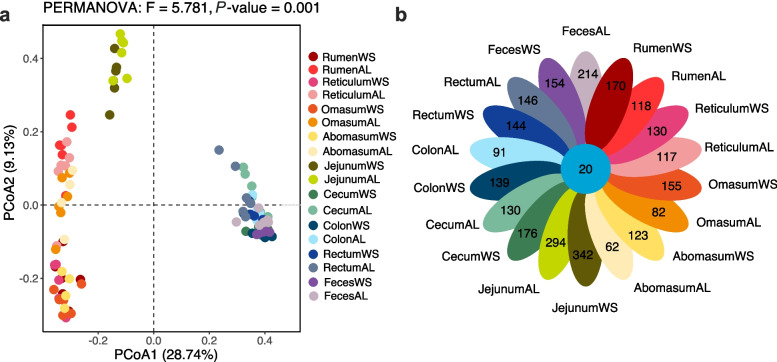
Beta diversity and ASVs composition analysis. **a** PCoA of gut microbes using Bray–Curtis dissimilarity values (PERMANOVA; *P* = 0.001). **b** Venn diagram for the bacterial ASVs in each part of GITs. WS, wheat straw group; AL, alfalfa group

Based on the principal co-ordinates analysis, shifting roughages had less contribution to the bacterial community than the spatial distributions in the major parts of GITs (Fig. 2a). This shows that the microbes of each GIT are mainly compartmentalized in the forestomach (rumen, reticulum, omasum, and abomasum), small intestine (jejunum) and hindgut (cecum, colon, and rectum), and the effect of feed composition differentiation is not obvious. Shifting roughages from AL to WS caused more independent ASVs in GITs of sheep, especially in the forestomach (Fig. S2). Compared with the other GITs, the jejunum had the greatest number of unique ASVs, with 342 independent ASVs in the WS group and 294 in the AL group, and there were only 20 ASVs shared in each group (Fig. 2b).

### Compositions of the bacteria community in GITs

**Fig. 3:**
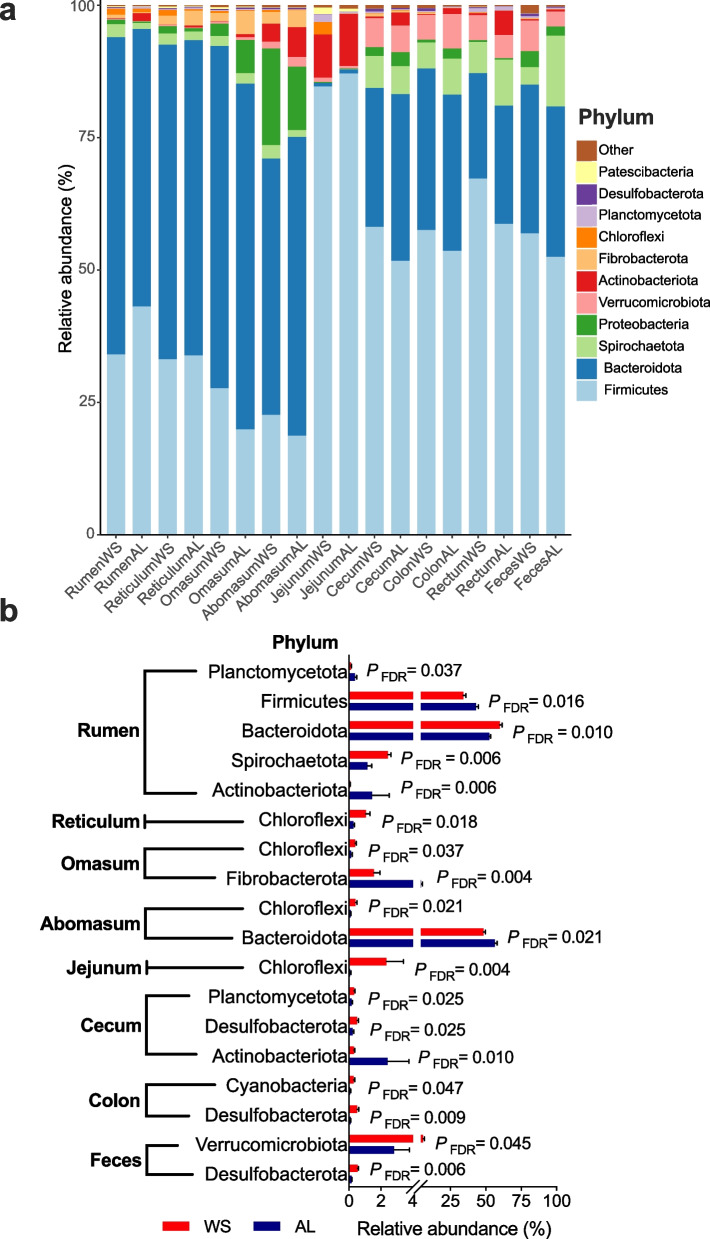
Bacterial phylum profiles in the gastrointestinal tracts of Hu sheep. **a** relative abundances of bacterial phyla; **b** differential bacterial phyla between treatments based on the analysis of linear discriminant analysis (LDA) effect size (relative abundance > 1%, LDA > 2, FDR adjusted *P* < 0.05). WS, wheat straw group; AL, alfalfa group

Figure 3a showed that Bacteroidota was the predominant phylum in the bacteria community of forestomach (48.37%-65.31%), whereas Firmicutes was the most common phylum in the jejunum (84.64%-87.06%), hindguts, and feces (51.70%-67.23%) of sheep. Shifting roughages from AL to WS significantly increased the relative abundances of Bacteroidota, Spirochaetota (*P*_*FDR*_ < 0.05), and decreased the relative abundances of Firmicutes, Actinobacteriota and Planctomycetota at phylum levels in the rumen of sheep (*P*_*FDR*_ < 0.05, Fig. 3b). It also significantly increased the relative abundance of Chloroflexi in the reticulum, omasum, abomasum and jejunum (*P*_*FDR*_ < 0.05), and the relative abundance of Desulfobacterota in the cecum, colon, and feces (*P*_*FDR*_ < 0.05). Besides, shifting roughages from AL to WS decreased the cecal Actinobacteriota (*P*_*FDR*_ = 0.010).

**Fig. 4:**
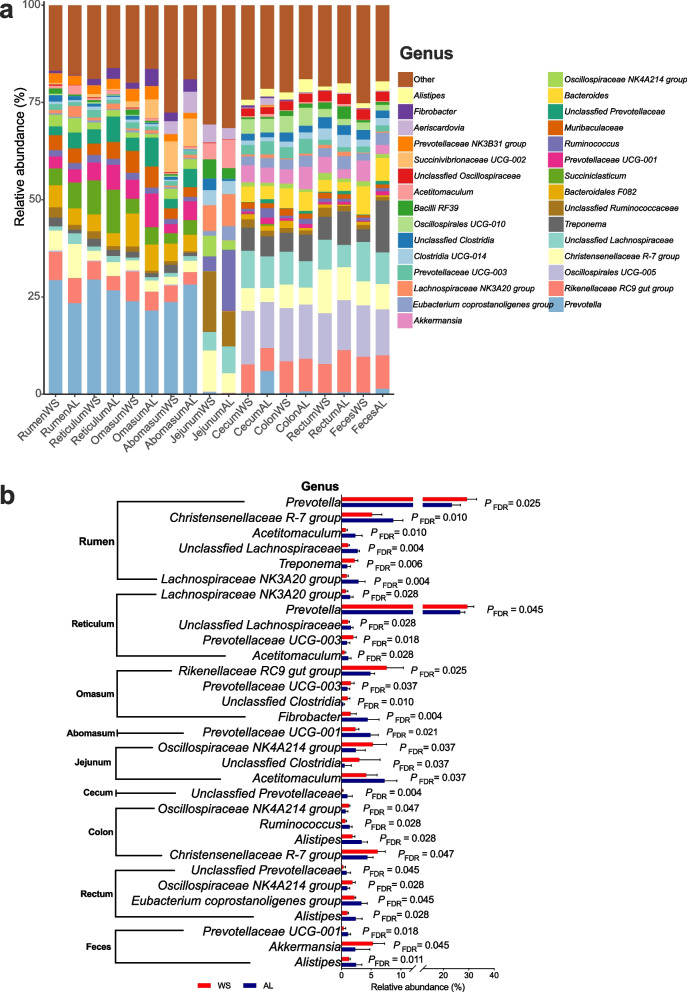
Bacterial genus profiles in the gastrointestinal tracts of Hu sheep. **a** relative abundances of bacterial genera; **b** differential bacterial genera between treatments based on the analysis of linear discriminant analysis (LDA) effect size (relative abundance > 1%, LDA > 2, FDR adjusted* P* < 0.05). WS, wheat straw group; AL, alfalfa group

At the genus level, *Prevotella* was the predominant bacteria in the rumen, reticulum, omasum, and abomasum (Fig. 4a). Shifting roughages from AL to WS increased the *Prevotella* relative abundance in the rumen and reticulum (*P*_*FDR*_ < 0.05), but decreased the relative abundances of *Lachnospiraceae NK3A20 group* and Unclassified Lachnospiraceae in rumen and reticulum (*P*_*FDR*_ < 0.05) (Fig. 4b). The predominant bacterial genus in jejunum were different between the WS group (*Unclassified Ruminococcaceae*) and AL group (*Ruminococcus*), while the predominant bacterial genus in the cecum, colon, rectum, and feces of sheep were *Oscillospiraceae UCG-005*. Meanwhile, shifting roughages from AL to WS increased the *Oscillospiraceae NK4A214 group* in the jejunum, colon, and rectum, and decreased the *Alistipes* in the colon, rectum, and feces of sheep (*P*_*FDR*_ < 0.05).

### Co-abundance network of bacterial community in ruminal contents and feces

**Fig. 5:**
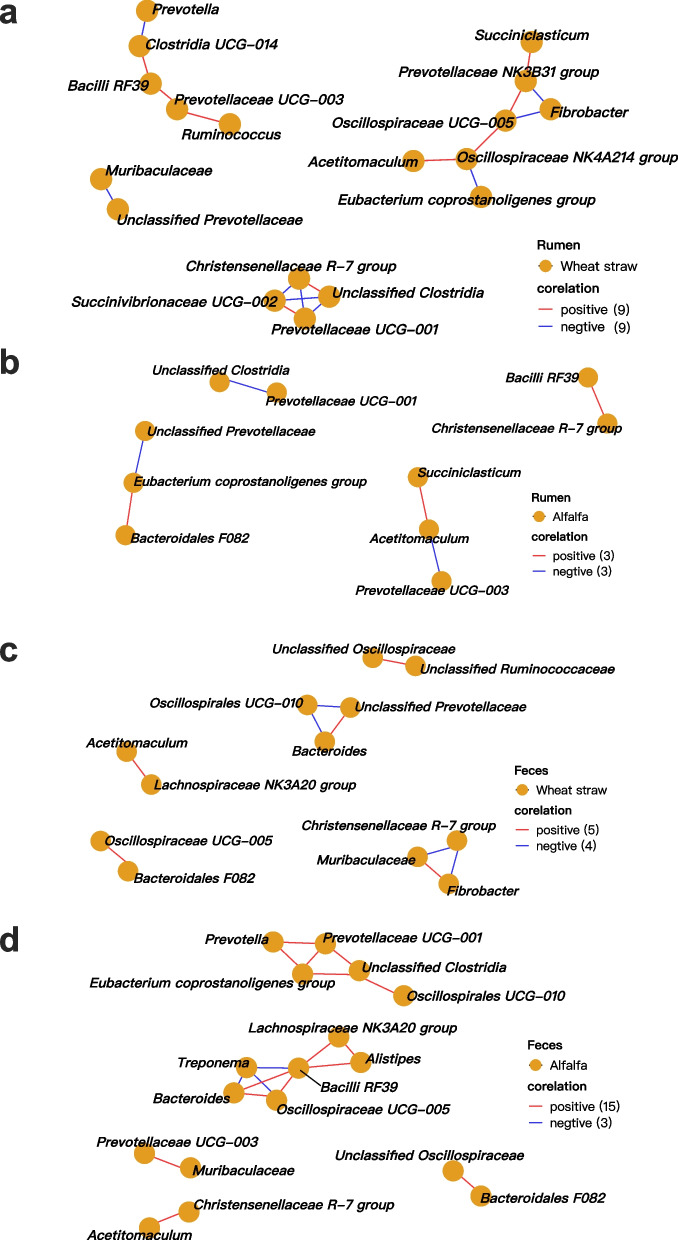
Co-abundance networks of the bacterial community in the rumen and feces of Hu sheep. Only the co-abundance with spearman correlation coefficient > 0.8, *P* < 0.05 were illustrated in the network; the circles represent bacterial genera

Bacterial networks achieved by the Spearman correlation analysis in rumen and feces were selected to represent the forestomach and hindgut based on the differential bacterial community at genus levels (Fig. 5). Shifting roughages from AL to WS caused more complex networks between rumen genera. A total of 18 co-abundances were found in the rumen bacterial community of sheep fed WS with 9 positive and 9 negative co-abundances, while just 3 positive and 3 negative co-abundances were identified in sheep fed AL. The negative co-abundance between *Prevotellaceae UCG-001* and *Unclassified Clostridia* was found in the rumen of both treatments*.* For dietary WS, ruminal genera belonging to Firmicutes had the most co-abundances (Fig. 5a). For example, the relative abundance of *Prevotella* had a negative correlation with *Clostridia UCG-014.* The *Christensenellaceae R-7 group* had a positive correlation with *Unclassified Clostridia* and negative correlations with *Succinivibrionaceae UCG-002* and *Prevotellaceae UCG-001*. However, ruminal genera belonging to Firmicutes and Bacteroidota showed negative correlations with each other in sheep fed AL (Fig. 5b), indicating a different rumen bacterial network compared with sheep fed WS.

The bacterial networks in sheep feces became simpler after shifting roughages from AL to WS (Fig. 5c, d), which differed from those in sheep rumen. A total of 9 co-abundances (5 positives and 4 negatives) between feces bacterial genera were obtained in sheep fed WS, while 18 co-abundances (15 positives and 3 negatives) were found in sheep fed AL. Feces bacterial genera belonging to Firmicutes showed the most co-abundances in the feces of both treatments. For example, *Alistipes* had positive correlations with *Bacilli RF39* and *Lachnospiraceae NK3A20 groups* (Fig. 5d), and *Bacilli RF39* had positive correlations with *Lachnospiraceae NK3A20 group*, *Bacteroides* and *Oscillospiraceae UCG-005*, but negatively correlated with *Treponema* in the feces of sheep fed wheat straw. For dietary AL, the relative abundance of *Treponema* had negative correlations with *Bacilli RF39*, *Bacteroides*, and *Oscillospiraceae UCG-005*, respectively.

### Prediction of the effect on the function profiles in different parts of GITs

Based on the predicted bacterial functions, shifting roughages from AL to WS increased the carbohydrate metabolism in the rumen and reticulum, and decreased the energy metabolism in the rumen and abomasum (*P*_*FDR*_ < 0.05, LDA score > 2) (Fig. S3). Meanwhile, it decreased the metabolism of cofactors and vitamins and lipid metabolism in the rumen as well as reticulum, omasum, and jejunum, respectively (*P*_*FDR*_ < 0.05, LDA score > 2). Notably, no significant difference was found in the predicted metabolic pathways of the bacterial community in the hindguts (cecum, colon, and rectum) of sheep (*P*_*FDR*_ > 0.05).

**Fig. 6:**
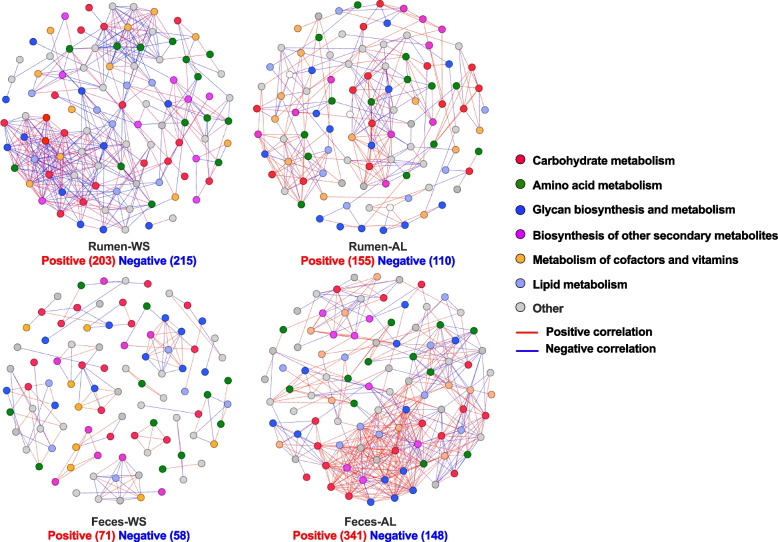
Co-abundance networks of predicted metabolic pathways in the rumen and feces of Hu sheep. Only the co-abundance with spearman correlation coefficient > 0.8, *P* < 0.05 were illustrated in the network. Circles represent the KEGG pathways at level 3. WS, wheat straw group; AL, alfalfa group. All the KEGG identifiers were from (http://www.kegg.jp/kegg/kegg1.html) [31–33]

The transition of roughages from AL to WS increased the complexity of the rumen metabolic pathway network, while the pathway network in the sheep feces became simpler (Fig. 6), which was similar to the bacterial networks. The pathways of carbohydrate metabolism, amino acid metabolism, and glycan biosynthesis and metabolism had the most contribution to the pathway networks in the rumen or feces of both treatments. A total of 203 positive and 215 negative pathway co-abundances were identified among ruminal bacteria of sheep fed wheat straw, while dietary AL caused 155 positive and 110 negative pathway co-abundances among ruminal bacteria.

Among level-3 pathways within carbohydrate metabolism, amino sugar and nucleotide sugar metabolism had the greatest positive correlations with other pathways, while pyruvate metabolism showed the most negative correlations with others in the rumen of sheep fed WS. Unlike feeding WS, the pathways of butanoate metabolism and pentose phosphate pathway were respectively the most linked nodes for positive and negative correlations in the rumen of sheep fed AL. The transition of roughages from AL to WS decreased the bacterial pathway co-abundances of bacterial metabolism at KEGG level-3 in sheep feces (489 vs. 129). The top three ranking of node numbers in the feces of both treatments were the same as the rumen bacterial network in sheep fed AL. Within carbohydrate metabolism pathways at level 3, the pathways of pyruvate metabolism and pentose phosphate pathway were respectively the most linked nodes for positive and negative correlations in the feces of sheep fed WS, while the starch and sucrose metabolism and the carbohydrate digestion and absorption were those nodes in the feces of sheep fed AL.

## Discussions

Roughages are the main feed and fundamental nutrients for ruminants, and the ability of ruminants to degrade the roughage is endowed by the function of microbiota in GITs [34]. Results of the present study showed that the transition from AL to WS mainly caused responses in rumen microbiota even under the isocaloric and isonitrogenous diets. The most dramatic changes in rumen microbiota structure were observed together with the predicted functions of carbohydrate metabolism, which were caused by the nutrient availability of roughages [35]. Typically, more than 50% of the forage is digested in the rumen. However, the untreated WS has a more recalcitrant structure rich in structural carbohydrates, which may make Hu sheep GITs employ more species for microbial co-degradation [36]. Previous results revealed that the increased NDF in the diet would decrease the circulation rate of indigestible NDF in the rumen, which increased the whole rumen weight [37]. These results were in agreement with our results that the rumen weights of sheep fed WS were greater than AL. The high content of NDF in the WS suppressed the passage rate of the feed in the rumen, resulting in the retention of coarse material. The low nutrient availability of WS enriched greater abundances of fiber-degrading bacteria, such as *Treponema* [35]. In addition, the differential bacteria communities in the reticulum and omasum were similar to those in the rumen after the transition of low-quality wheat straw. The reason could be that these GITs are the major sites for microbial digestion of dietary crude fiber in host animals [38]. Results of the present experiment also indicated that microbes of each GITs were compartmentalized in the forestomach, small intestine, and hindguts, rather than differentiated by forage composition, which was consistent with previous findings [39].

The GITs function is inextricably linked to microbiota composition, hence, there is a need for a better understanding and a thorough analysis of those processes. At the genus level, the relative abundance of *Prevotella* was significantly increased in the rumen and reticulum in the WS group compared to the AL group. Members of *Prevotella* have glycolytic potential and could metabolize sugars to acetate, propionate, and succinate [34]. Therefore, the increased relative abundance of *Prevotella* after feeding WS was inseparable from the enhanced carbohydrate metabolism in the rumen and reticulum. Meanwhile, previous results indicated that this genus is also involved in glycan biosynthesis [40]. However, more *Prevotella* is involved in the degradation of refractory wheat straw in the forestomach, also increasing the functional redundancy of these GITS, thereby reducing the efficiency of degradation.

It is worth noting that the Unclassified Lachnospiraceae and *Lachnospiraceae NK3A20 group* was decreased in the rumen and reticulum of the WS group in the present experiment. Some members of this family can protect against inflammation by producing butyrate [41]. Therefore, the lower abundance of this genus in the WS group implies that feeding WS may cause potential damage to animal health. Similarly, the WS group had a significant reduction of *Prevotellaceae UCG-001* in the abomasum and feces, as well as *Alistipes* in the colon, rectum, and feces, compared to the AL group. Some studies have found that *Prevotellaceae UCG-001* is negatively correlated with markers of glucose and lipid metabolism disorders [42], and *Alistipes* is also an important genus of anti-inflammatory bacteria [43]. The decrease of these bacterial genera possibly increased the inflammation levels in the GITs of sheep fed WS, thereby affecting their growth performance. Besides, feeding WS significantly reduced the lipid metabolism of jejunal bacteria, which would further affect the lipid deposition and absorption in the small intestine of sheep [44, 45].

Carbohydrate metabolism and amino acid metabolism are major bacterial functions in all GITs. Results of the present experiment found significant differences in these pathways between treatments in the rumen and feces of sheep. Generally, 70 to 85% of the digestible dry matter in the diet can be fully fermented and digested by microbiota in the rumen, and the whole process involves a rigorous carbohydrate metabolism process [46], while feces are generally hindgut representatives in microbiological research [47], therefore a more detailed analysis of rumen and feces. Feed nutrient composition is related to the type of fermentation in the rumen [34], with feeds with higher fiber content producing lower levels of propionate and butyrate [48]. Thus, most metabolic pathways of rumen bacteria had positive correlations with the ruminal butyrate concentration in sheep fed AL. The decreased correlations between the pyruvate metabolism pathway and other pathways after shifting roughages to WS would reduce the precursor for various volatile fatty acids [48], and consequently affect the dietary energy utilization in ruminants. The pentose phosphate pathway is closely related to gluconeogenesis and was the most negatively related metabolism in the feces after feeding WS in the present experiment. This result indicated that the harmful impacts of WS on bacterial energy metabolism is not only limited to the forestomach but also extended to the hindguts.

Opposite results were found in the co-abundance networks of bacterial communities and functions between bacterial communities in the rumen and feces of sheep in the present experiment. The transition of roughage from AL to WS increased the microbial diversity of the rumen microbiota and complicated the microbial and metabolic network. The increased diversity caused by WS would further result in the functional redundancy of rumen bacteria, especially the fiber-degrading bacteria belonging to the genus *Prevotella*. However, nutrients would be preferentially used by the excess rumen bacteria and their redundant functions rather than the host animals, which thereby reduced the utilization efficiency of dietary energy [49]. This agreed with our results that feeding WS reduced the energy metabolism of the rumen bacterial community.

After digesting in the forestomach and small intestine, the roughage residues would enter the hindgut and be utilized by the hindgut microbes. Unlike AL, WS could not provide enough digestible material to satisfy hindgut microbial degradation, which caused the simplified networks of hindgut microbes and their metabolic pathways. Despite less diversity of hindgut bacteria would improve the efficiency of energy metabolism [47], the nutrient utilization in sheep fed WS was still limited and not as good as AL. Our study found that even under isocaloric and isonitrogenous diets, the transition of roughage from AL to WS still caused significant changes in the Hu sheep microbiota in GITs, which affects the bacteria function and degradation effect of the GITs. The reason for this phenomenon is inseparable from the refractory structural carbohydrate composition of wheat straw, resulting in low nutrient availability. However, although our study has analyzed the relationship between GITs and function, and inferred the effect of feeding WS on the growth performance of Hu sheep, the KEGG metabolic pathway is only obtained through prediction. In the follow-up, it is necessary to measure the nutrient content of the two diets after they reach the intestinal tract after digestion, which is helpful to further understand the impact of WS feeding on Hu sheep. Appropriate physical, chemical, and biological pretreatment of straw is worth considering if we want to further improve the availability of WS feeding in roughage substitution. The destruction of the cross-linked state within structural carbohydrates would improve the palatability and nutrient availability of WS, which can make it more suitable for feed utilization [50, 51].

## Conclusions

In conclusion, our results showed that feeding Hu sheep with untreated WS instead of alfalfa as roughage caused changes in the bacterial community in the GITs of Hu sheep, even under the isocaloric and isonitrogenous diet conditions. This alteration of the bacterial community is distinct in the forestomach and hindgut and mainly in the rumen. Microbiota diversity and functional analyzes found that feeding untreated WS might reduce the abundance of anti-inflammatory bacteria in the whole GITs and potentially affect fatty acid synthesis. Further studies need to focus on the pretreated straw and the suitable ratio of straw to alfalfa in the feed to understand the role of straw replacing alfalfa in ruminant feed utilization.

## Supplementary Information

**Additional file 1:  Table S1. **The results of different parts of weight of Hu sheep gastrointestinal tracts. **Figure S1.** Rarefaction curves at 100% similarity for each treatment.** Figure S2.** Venn diagram of ASVs in each gastrointestinal tract. WS, wheat straw group; AL, alfalfa group.** Figure S3.** Level 2 KEGG pathway predictions in different groups. KEGG pathway with significant differences (LDA > 2), results are presented when proportion >1%, FDR adjusted* P* < 0.05. WS, wheat straw group; AL, alfalfa group. All the KEGG identifiers were from (http://www.kegg.jp/kegg/kegg1.html) .

## Data Availability

The datasets supporting the findings of this study are available in the NCBI Sequence Read Archive (SRA) database (Accession Number: PRJNA861451), https://www.ncbi.nlm.nih.gov/sra/PRJNA861451.

## Abbreviations

GITs: Gastrointestinal tracts
WS: Wheat straw
AL: Alfalfa hay
NDF: Neutral detergent fiber
ADF: Acid detergent fiber
LDA: Linear discriminant analysis
LEfSe: LDA effect size
KEGG: Kyoto Encyclopedia of Genes and Genomes
PICRUSt2: Phylogenetic Investigation of Communities by Reconstruction of Unobserved States 2
SEM: Standard error of the mean
