# Supplementary material for: Nutrient availability of roughages in isocaloric and isonitrogenous diets alters the bacterial networks in the whole gastrointestinal tract of Hu sheep
Source: BMC Microbiol. 2023 Mar 15;23:70. doi: 10.1186/s12866-023-02814-z (PMC10015938; doi:10.1186/s12866-023-02814-z)

**Additional file 1**

**Nutrient availability of roughages in isocaloric and isonitrogenous diets alters the bacterial networks in the whole gastrointestinal tract of Hu sheep**

Yuqi Li^a†^, Jian Gao^a†^, Yihan Xue^a^, Ruolin Sun^a^, Xiaoni Sun^a^, Zhanying Sun^a^, Suozhu Liu^b^, Zhankun Tan^b^, Weiyun Zhu^a^, Yanfen Cheng^a^*

^a^ Laboratory of Gastrointestinal Microbiology, National Center for International Research on Animal Gut Nutrition, Nanjing Agricultural University, Nanjing 210095, China

^b^ College of Animal Science, Tibet Agricultural and Animal Husbandry University, Nyingchi 860000, China

^†^These authors contributed equally to this work.

**Supplementary Table.**

**Table S1.** The results of different parts of weight of Hu sheep gastrointestinal tracts

| Items | Full weight (kg) | | *P* value |
| --- | --- | --- | --- |
|  | Alfalfa | Wheat straw |  |
| Rumen | 5.250 ± 0.140 | 8.370 ± 0.430 | <0.01 |
| Reticulum | 0.318 ± 0.042 | 0.373 ± 0.039 | 0.390 |
| Omasum | 0.290 ± 0.026 | 0.328 ± 0.058 | 0.580 |
| Abomasum | 0.580 ± 0.010 | 0.578 ± 0.097 | 0.990 |
| Jejunum | 1.510 ± 0.060 | 1.460 ± 0.070 | 0.580 |
| Cecum | 0.465 ± 0.060 | 0.488 ± 0.039 | 0.760 |
| Colon | 1.21 ± 0.07 | 1.13 ± 0.08 | 0.530 |
| Rectum | 0.195 ± 0.013 | 0.132 ± 0.007 | 0.010 |

**Supplementary Figures.**

**Figure S1.** Rarefaction curves at 100% similarity for each treatment.


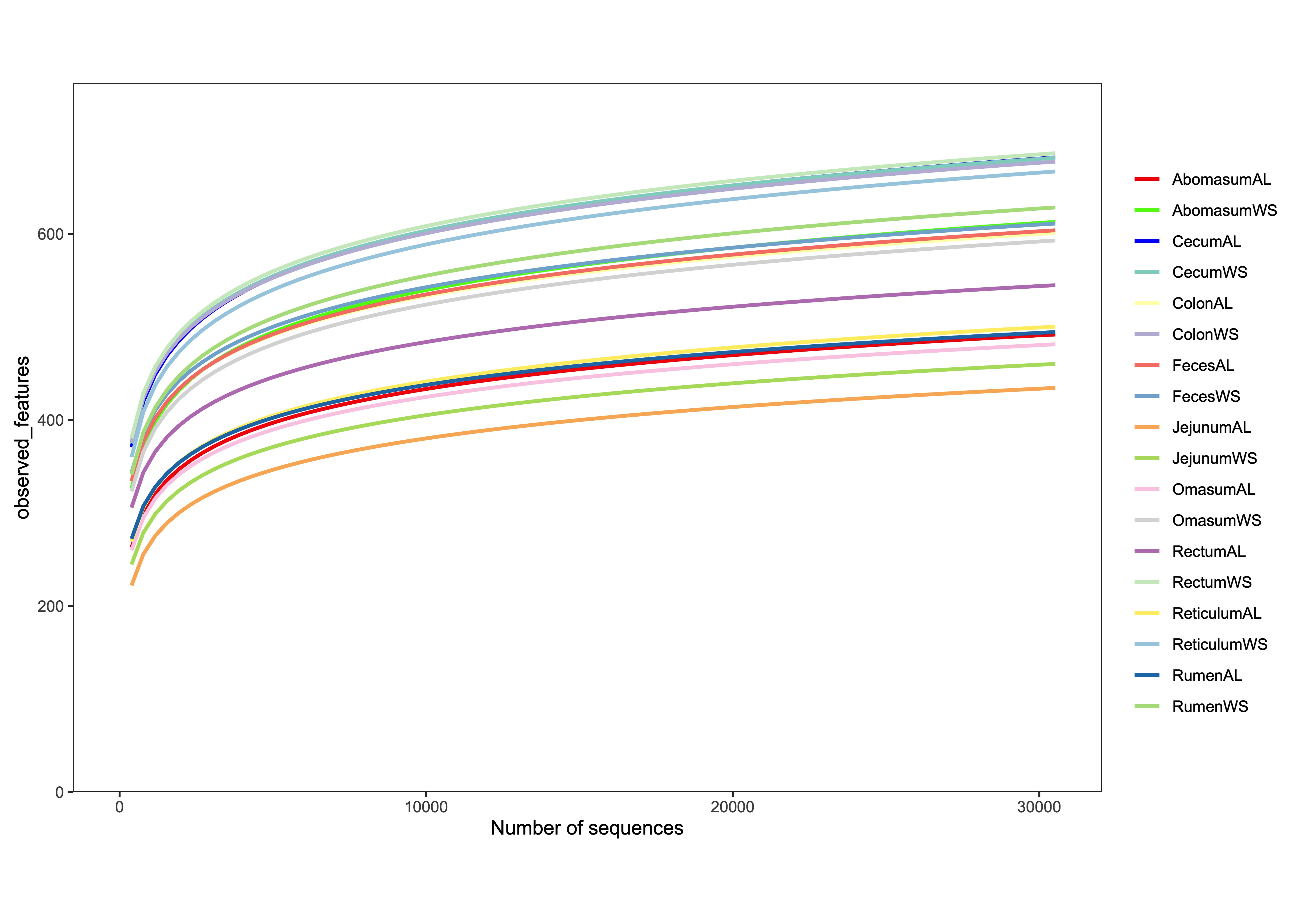


**Figure S2.** Venn diagram of ASVs in each gastrointestinal tract. WS, wheat straw group; AL, alfalfa group.


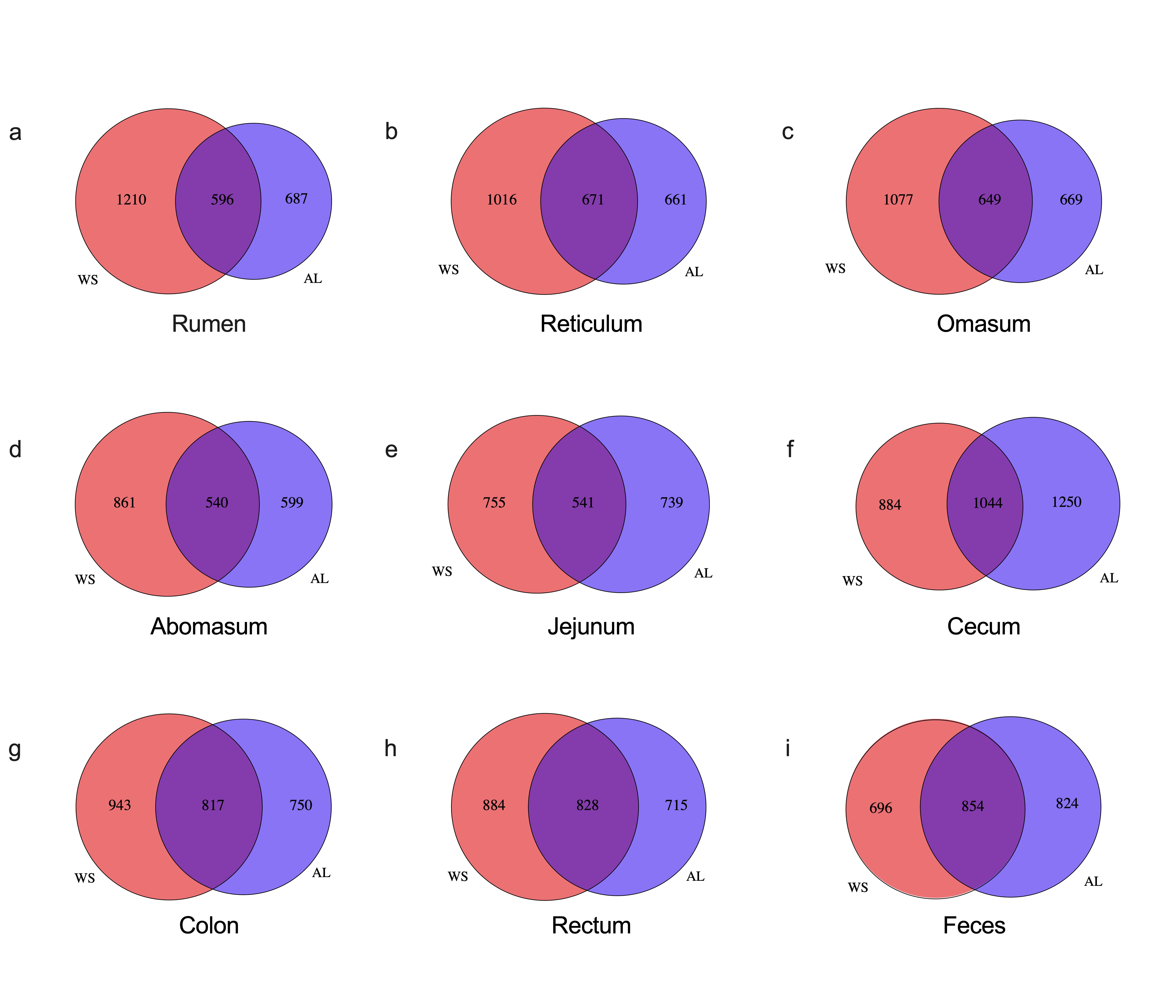


**Figure S3.** Level 2 KEGG pathway predictions in different groups. KEGG pathway with significant differences (LDA > 2), results are presented when proportion >1%, FDR adjusted *P* < 0.05. WS, wheat straw group; AL, alfalfa group. All the KEGG identifiers were from (www.kegg.jp/kegg/kegg1.html).


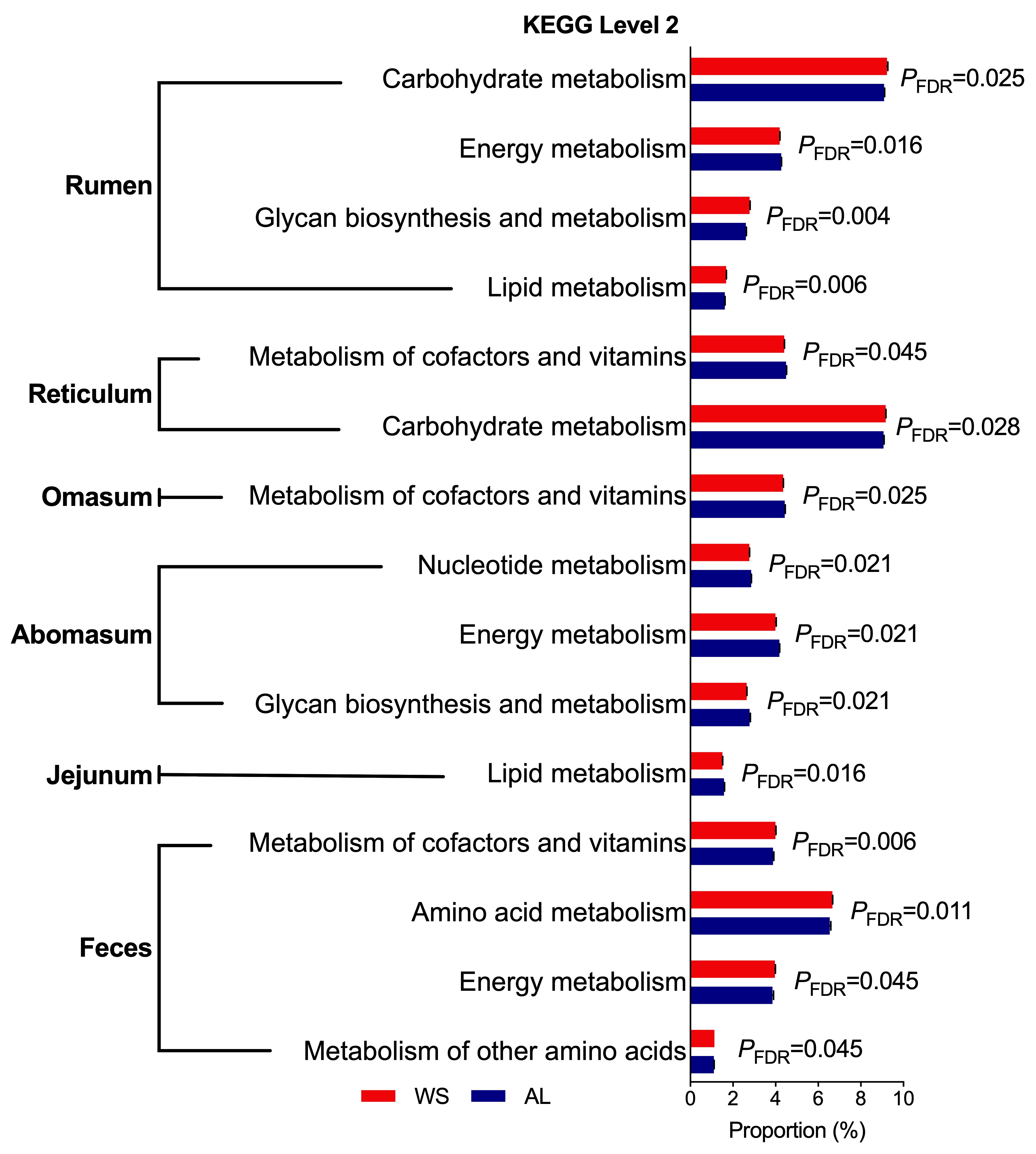

Supplement: Supplementary file 1 — Additional file 1: Table S1. The results of different parts of weight of Hu sheep gastrointestinal tracts. Figure S1. Rarefaction curves at 100% similarity for each treatment. Figure S2. Venn diagram of ASVs in each gastrointestinal tract. WS, wheat straw group; AL, alfalfa group. Figure S3. Level 2 KEGG pathway predictions in different groups. KEGG pathway with significant differences (LDA > 2), results are presented when proportion >1%, FDR adjusted P < 0.05. WS, wheat straw group; AL, alfalfa group. All the KEGG identifiers were from (http://www.kegg.jp/kegg/kegg1.html) . [file 12866_2023_2814_MOESM1_ESM.docx]
